# Metagenomic classification with KrakenUniq on low-memory computers

**DOI:** 10.21105/joss.04908

**Published:** 2022-12-28

**Authors:** Christopher Pockrandt, Aleksey V. Zimin, Steven L. Salzberg

**Affiliations:** 1 Center for Computational Biology, Johns Hopkins University, Baltimore, MD 21218, USA; 2 Department of Biomedical Engineering, Johns Hopkins University, Baltimore, MD 21218, USA; 3 Department of Computer Science, Johns Hopkins University, Baltimore, MD 21218, USA; 4 Department of Biostatistics, Johns Hopkins University, Baltimore, MD 21218, USA

## Abstract

Kraken and KrakenUniq are widely-used tools for classifying metagenomics sequences. A key requirement for these systems is a database containing all *k-mers* from all genomes that the users want to be able to detect, where *k* = 31 by default. This database can be very large, easily exceeding 100 gigabytes (GB) and sometimes 400 GB. Previously, Kraken and KrakenUniq required loading the entire database into main memory (RAM), and if RAM was insufficient, they used memory mapping, which significantly increased the running time for large datasets. We have implemented a new algorithm in KrakenUniq that allows it to load and process the database in chunks, with only a modest increase in running time. This enhancement now makes it feasible to run KrakenUniq on very large datasets and huge databases on virtually any computer, even a laptop, while providing the same very high classification accuracy as the previous system.

**Statement of need:**

The KrakenUniq software classifies reads from metagenomic samples to establish which organisms are present in the samples and estimate their abundance. The software is widely used used by researchers and clinicians in medical diagnostics, microbiome and environmental studies.

Typical databases used by KrakenUniq are tens to hundreds of gigabytes in size. The original KrakenUniq code required loading the entire database in RAM, which demanded expensive high-memory servers to run it efficiently. If a user did not have enough physical RAM to load the entire database, KrakenUniq resorted to memory-mapping the database, which significantly increased run times, frequently by a factor of more than 100. The new functionality described in this paper enables users who do not have access to high-memory servers to run KrakenUniq efficiently, with a CPU time performance increase of 3 to 4-fold, down from 100+.

## Introduction

The GenBank genome repository (Benson et al., 2012) currently contains over 400,000 prokaryotic genomes and over 20,000 eukaryotes, including thousands of microbial eukaryotes such as fungi and protists. To take advantage of this ever-growing variety of microbial sequences, metagenomic sequence analysis methods must create customized databases that capture all of this sequence diversity. Tools such as Kraken (Wood & Salzberg, 2014) and KrakenUniq (Breitwieser et al., 2018) classify DNA or RNA sequencing reads against a pre-built database of genomes using an exact *k-mer* matching strategy that is not only highly accurate but that, because it avoids the step of sequence alignment, makes both systems extremely fast.

The Kraken database is a customized, compressed data structure that associates a unique taxonomy identifier with every single *k-mer* in every genome in the database. (Note that both Kraken and KrakenUniq use the same database design, so we will refer to both as Kraken databases.) If a *k-mer* occurs in two or more genomes, then the database stores the taxonomy ID associated with the lowest common ancestor of those genomes. This strategy means that only a single ID is attached to each *k-mer*.

However, with the number of genomes available today, a standard Kraken database will contain billions of *k-mers*, and even with careful compression this data structure can grow very large. A key requirement for the speed of the Kraken algorithm (which is 900 times faster than MegaBlast (Wood & Salzberg, 2014)) is the loading of the entire database into main memory. For the large databases and read datasets that are commonly used in metagenomics experiments today, this requires dedicated machines with large amounts of RAM (e.g., exceeding 100 GB or even 400 GB), without which classification becomes slow and impractical. The newer Kraken2 system (Wood et al., 2019) achieves a significantly lower memory footprint by using probabilistic data structures to reduce the database size, at the cost of slightly lower accuracy than KrakenUniq. This reduction in accuracy includes a very small but non-zero false positive rate (i.e., where the system incorrectly reports that a *k-mer* is present in a particular genome), which is problematic for certain applications that require very high precision. In particular, when metagenomic sequencing is used for the diagnosis of infections in a clinical setting (Salzberg et al., 2016), the pathogen of interest might be detected from just a handful of reads. In that scenario, even a few false positives can be confusing, and KrakenUniq is the preferred method rather than Kraken2.

By default, KrakenUniq performs memory mapping to load the database; i.e., it does not load the entire database into main memory. (Kraken 1 employs the same strategy.) This makes classification of larger read datasets much slower, but it allows KrakenUniq to run on machines with low available main memory. If enough free RAM is available to hold the entire database in main memory, users are recommended to explicitly load the entire database prior to classification using the flag --preload, which dramatically speeds up the classification (see Table 1).

To improve KrakenUniq’s performance when not enough main memory is available to load the entire database into RAM, we have added a new capability to KrakenUniq, which we call database chunking. This new feature is released in KrakenUniq v1.0.0 and subsequent releases at: https://github.com/fbreitwieser/krakenuniq (Github) https://anaconda.org/bioconda/krakenuniq (Conda)

### Database chunking

The KrakenUniq database consists of two tables: A *k-mer* table maps each *k-mer* to its taxonomic ID and is sorted by the *k-mers’* minimizers (Roberts et al., 2004). A second table, the minimizer table, is lexicographically sorted and maps each minimizer to the corresponding *k-mers* in the *k-mer* table which form a contiguous block. Hence, the database can be chunked by taking a chunk of the minimizer table and the corresponding range of the *k-mer* table that contains all *k-mers* for the selected minimizers.

Under this new algorithm, KrakenUniq loads one chunk of the database into memory at a time. KrakenUniq performs a binary search on the minimizer table to find the largest minimizer such that the chunk of the minimizer table and the corresponding chunk of the *k-mer* table together use not more than the specified amount of memory. It then loads the chunk of the minimizer table and the corresponding chunk of the k-mer table and iterates over all of the reads provided as input. The code looks up all *k-mers* in the reads in the currently-loaded chunk of the database. The taxonomic IDs for the *k-mers* that matched a k-mer in the chunk (or placeholders for *k-mers* without a hit) are stored in a temporary file on disk for each read. With every chunk iteration, taxonomic IDs of newly found *k-mers* in the reads are appended to the temporary file. This process is repeated until the entire database has been processed. At this point the temporary file contains taxonomic IDs for all k-mers in the reads that matched any part of the database. KrakenUniq then reads the temporary file, collects *k-mer* hits for every read, and uses them to classify each read. Classification results will be identical to running in the default mode; i.e., database chunking does not alter the output.

This new feature makes it feasible to run KrakenUniq on very large datasets and huge databases on virtually any computer, even a laptop, while providing exact classifications that are identical to those of KrakenUniq in its other modes. Users can employ this feature by using --preload-size to specify the amount of available main memory that they want to use for loading chunks of the database, e.g., --preload-size 8G or --preload-size 500M.

Running times can vary significantly depending on the type of storage (e.g., databases on network storage can take longer to load) and the size of the read dataset (i.e., classifying a small number of reads does not justify preloading the entire database, especially of fast storage). The speed of loading the database is not impacted by the --preload-size option because the database is still read in a linear way.

Saving intermediate files from the chunks is done in the same way as in the original code. The only difference is that now classification results from all individual chunks are concatenated into a single file, which is read once all chunks are finished. Table 1 shows that in a typical use case, even when the database does fit in RAM, loading the entire database (--preload option) is far faster than memory mapping (14 minutes versus 48 hours). Loading the database by chunks adds overhead because of the need to iterate over the reads multiple times, but is still comparable to pre-loading the entire database and highly recommended when not enough main memory is available. For example, limiting the database to 8G, which means it can be loaded even on a standard laptop computer, increased the running time only about 3.4-fold, even though the database was broken into 49 chunks. For large read datasets we expect that setting the --preload or --preload-size flag will always be faster than the default behavior of memory mapping. The format of the databases used by the new algorithm has not changed, hence all previously built databases for Kraken and KrakenUniq can be used.

This feature has only been added to KrakenUniq, and not to Kraken, which is no longer actively maintained. Because KrakenUniq offers more features, shares the same implementation with Kraken and produces the same output, we highly recommend that users upgrade from Kraken to KrakenUniq.

## Figures and Tables

**Table 1:** Running times for classifying 9.4 million reads (from a human eye metagenome, accession SRR12486990, available from NCBI at https://www.ncbi.nlm.nih.gov/sra/SRR12486990) with 8 threads using KrakenUniq in different modes. The database size was 392 GB, and it consisted of all complete bacterial, archeal, and viral genomes in RefSeq from 2020, 46 selected eukaryotic human pathogens (Lu & Salzberg, 2018)), as well the human genome and a collection of common vector sequences. The database is available for download at https://benlangmead.github.io/aws-indexes/k2 under the name EuPathDB46. The command lines used to measure the runtimes were krakenuniq --db krakendb-2020–08-16-all_pluseupath --threads 24 --report-file report --output classify SRR12486990.fastq with no additional options for default, and with addition of the preload option shown in the table for various preload sizes. in the database chunking experiments (using –preload-size) KrakenUniq loaded the database into RAM in 49, 25, 13 and 7 chunks (respectively).

Mode	Running time
default (memory mapping)	48 hours
--preload	14 min
--preload-size 8G	47 min
--preload-size 16G	32 min
--preload-size 32G	25 min
--preload-size 64G	19 min
